# Spending Time with Mothers as a Resource for Children with Chronic Diseases: A Comparison of Asthma, Type 1 Diabetes, and Cancer during COVID-19 Pandemic

**DOI:** 10.3390/ijerph192114126

**Published:** 2022-10-29

**Authors:** Silvia Spaggiari, Virginia Forlini, Silvia Carraro, Valentina Agnese Ferraro, Stefania Zanconato, Maria Montanaro, Valerio Cecinati, Silvana Zaffani, Claudio Maffeis, Daniela Di Riso

**Affiliations:** 1 Department of Developmental and Socialization Psychology (DPSS), University of Padua, 35131 Padova, Italy; 2 Department of Women’s and Children’s Health, University of Padova, 35128 Padova, Italy; 3 Complex Structure of Pediatrics and Pediatric Oncohematology “Nadia Toffa”, Central Hospital Santissima Annunziata, 74121 Taranto, Italy; 4 Pediatric Diabetes and Metabolic Disorders, Department of Surgical Sciences, Dentistry, Paediatrics and Gynaecology, University of Verona, 37134 Verona, Italy

**Keywords:** time spent with mothers, chronically ill children, COVID-19 pandemic

## Abstract

Due to the COVID-19 pandemic, many families had to manage new difficulties, especially those of chronically ill children. More and more research has focused on the negative effects of the pandemic on psychological wellbeing, while less is known about the resources. The present study aimed to explore the role of time spent with mothers in chronically ill children’s populations during the COVID-19 pandemic. Moreover, it explored the differences in mothers’ and children’s psychosocial functioning in three clinical populations. Four groups were recruited and compared: 7–15 year old children with asthma (45), type 1 diabetes (52), and cancer (33), as well as their healthy counterparts (41), and their respective mothers. They were administered standardized questionnaires and ad hoc surveys assessing psychological wellbeing and worries. Children of the four groups scored significantly differently with respect to the concerns for contagion, internalizing symptoms, and prosocial behaviors; mothers had worries about the consequences of their children’s contagion related to the chronic illness, as well as time with the child. The multiple linear regression model showed an association of being affected by cancer, suffering from type 1 diabetes, and spending less time with the child with an increase in children’s internalizing problems. Time with mothers seemed to be a resource for psychological wellbeing during the pandemic. Clinical implications are discussed.

## 1. Introduction

For the last 2 years, the world has been struggling a hard fight against the COVID-19 pandemic. In 2020, Italy was one of the main affected countries in the world, according to the number of deaths due to COVID-19 contagion [1]. The government imposed a national lockdown, strict movement limitations, and closure of nonessential economic activities, services, and schools [2]; Phase 1 from 9 March, Phase 2 from 4 April, and Phase 3 from 18 May represented a gradual reopening and progressively softened movement restrictions [3]. Until then, people went through several waves of the disease in which safety rules were suspended or restored. The period immediately following the first lockdown, from May 2020 to September 2020, is called Phase 4 for the present paper’s aim. The situation in Italy in those months was of gradual switching to normality; people could move, many activities reopened, and social constraints were reduced, but the COVID-19 pandemic remained alarming [4].

The restrictions imposed changed the day-to-day life and routines of families. Children spent on average 30 h a week and 22 h a week, respectively, at school and on activities outside before the pandemic, which is time that they had to spend at home, during the first lockdown [5]. Parents needed to be with their children supervising or helping them with school duties, for most of this time. Thus, a significant increase in time spent with parents was evidenced [5]. Whether spending more time together is a risk factor or a resource is still unclear. On one hand, a recent study demonstrated that the increased amount of time spent with parents was considered one of the benefits perceived by children and adolescents in COVID-19 home quarantine [6]. Sharing, involvement in activities together, and communication were the most appreciated aspects [7]. On the other hand, due to school and workplace closure, parents had to take care of their children, with greater educational and supervision responsibilities, while trying to adapt to working from home; thus, time with parents may have been of poor quality [5]. When it comes to the chronically ill children population, time with parents may have played a crucial role, as it is managed differently. Changes in health service availability increased parents’ responsibility in the medical care of their children and had an impact on chronic conditions, e.g., on treatment adherence [8]. Thus, mothers may have had to spend more time giving more careful care and better monitoring their children’s disease; however, they may also have been more controlling and anxious with their children. To our knowledge, no studies explored the role of time spent with parents and, more specifically, mothers in chronically ill children’s populations during the COVID-19 pandemic. Moreover, several studies explored the vulnerability factors that contribute to determining the magnitude of the impact of the COVID-19 pandemic on children and their parents’ psychological wellbeing, while less is known about the protective factors [9]. The adoption of a “benefit finding” perspective, i.e., the identification of positive effects resulting from a trauma [10], was found among benefit factors against psychological experiences such as depression, anxiety, and stress [6]. Generally, benefit finding is typical in clinically ill children and adolescents affected by cancer or type 1 diabetes [11,12,13,14], and there are findings that it is negatively associated with mental health [15]. Adequate and clear communication between children and their parents [6], good quality of family relationships [16], maintenance of routines and rituals [16], physical activity [17], and keeping in contact with friends [17] were some of the resources for mental health during the pandemic as evidenced by the literature. It would be important to pay specific attention to the role of time with parents for chronically ill children during pandemic times, as it may be considered from a “benefit finding” perspective. Moreover, some studies claimed that there are biases based on children’s gender in the time that mothers and fathers spend with their daughters and sons in nonclinical samples. Actually, it seems that mothers tend to spend more time with their daughters, probably led by same-sex identification and greater similarities [18,19]. Whether these differences were significant during the pandemic has not yet been studied.

All mentioned studies refer to the lockdown. Time is a concept that has changed these last years, mainly due to the pandemic. No studies, in particular, explored the role of time during Phase 4, while it can be useful to analyze if time with mothers was a resource or a risk factor, in a period of shifting to normality, as we are living today, in order to inform prevention interventions.

What was more clearly evidenced by the literature is the detrimental effects of quarantine on people’s psychological wellbeing. A significant increase in anxiety and depression was mainly reported, alongside PTSD symptoms, boredom, stress, anger, irritability, and fear both for children, adolescents, and their parents [20,21]. Research predominantly focused on healthy populations, while less knowledge is available about the effects of the pandemic on chronically ill children and their caregivers [3,22,23]. Pediatric patients with chronic conditions are at higher risk for mental health problems compared to healthy peers, and this vulnerability may be even stronger during pandemic times [24]. On the other hand, it can be supposed that growing up in more challenging circumstances helps people become more resilient; thus, they may have more chances to maintain a psychological equilibrium during harsh times such as a pandemic [24]. A recent review evidenced that children with chronic physical conditions were more likely to have greater internalizing symptoms (mainly anxiety and depression) in comparison to their healthy peers during the COVID-19 pandemic [25], while other authors found no differences in internalizing problems between the two groups [24]. Other research concentrated on COVID-19-related fears, evidencing that children with asthma had higher concerns of contagion, but no differences in psychological wellbeing, if compared with healthy peers [3]. When it comes to parents, it seems clearer that the pandemic had a pejorative effect on their mental health; greater COVID-19 concerns related to them and their children [3,22] and higher risk for anxiety, fears, and stress have been reported compared to parents of healthy children [26,27].

Moreover, few studies focused on psychological functioning have been carried out comparing children affected by different chronic pathologies and their caregivers both in normal and in pandemic times. However, an illness-specific approach is fundamental for providing adequate care to these vulnerable populations [28]. Asthma, diabetes, and cancer, in particular, are very often considered in meta-analyses or reviews in the literature regarding the psychological functioning of children with chronic illnesses (e.g., Barlow and Ellard, 2004) [29]. Varni et al. (2007) reported that children with type 1 diabetes tend to show a higher health-related quality of life (HRQOL) compared to children with asthma, cancer, and gastrointestinal and cardiac conditions [30]. Hullman et al. (2010) found that parents of children with asthma perceived their children as more vulnerable than parents of children with type 1 diabetes or cancer [28]; moreover, caregivers of children with asthma and type 1 diabetes showed higher parenting stress if compared with parents of children with cancer [28]. To the best of our knowledge, no studies focused on the comparisons between children with different chronic illnesses and their parents, during Phase 4.

Considering the gaps in the literature, the present research objective was to explore the role of time spent with mothers on children with chronic diseases’ psychological wellbeing, during the early pandemic times. The study compared three clinical samples of children with asthma, type 1 diabetes, and cancer, and their mothers with a control group of healthy children and mothers, during Phase 4. Firstly, we aimed to investigate the worries related to the pandemic and the psychological general wellbeing of children with asthma, type 1 diabetes, and cancer, and their mothers during Phase 4. It was hypothesized that children with chronic diseases had greater fears related to COVID-19 and worse psychological outcomes if compared to their healthy peers [24,25]. As to mothers, their worries related to the pandemic, time with the child, and general psychological wellbeing were assessed. It was hypothesized that mothers of the clinical groups had greater fears related to COVID-19, spent more time with their children, and had worse psychological outcomes if compared to the control sample’s mothers [4,26,27]. Secondly, in order to identify psychological specificities for each chronic disease and encourage a disease-specific approach [28], an explorative comparison between children and mothers of the three conditions was made with respect to their worries related to COVID-19, their psychological wellbeing, and time with the child. Lastly, it was hypothesized that time with mothers is a resource in chronically ill populations [5,7]. A diagnosis of asthma, type 1 diabetes, and cancer [24], children’s gender [18,19], less time together [5,7], greater children’s worries for COVID-19 contagion [3], and worse mothers’ psychological wellbeing [26,27] are expected to be predictors of children’s internalizing symptoms for all three clinical groups.

## 2. Materials and Methods

### 2.1. Participants

Four different samples were considered: 45 children with asthma (77.8% males, 22.2% females), 52 children affected by type 1 diabetes (53.8% males, 46.2% females), 33 children with a diagnosis of cancer (48.5% males, 51.5% females), and 41 healthy children considered as the control sample (68.3% males, 31.7% females). The age range was between 7 and 15 years old, with no significant difference between the groups in age (see Table 1). A significant gender difference emerged between the groups with a prevalence of females for the cancer group and of males for the asthma group (see Table 1). As a medical index for the asthma group, the GINA test [31] was used; it is a clinician’s evaluation of asthma control on the basis of symptoms, need for relievers, limitation to physical activity, and spirometry parameters. The participants’ control of asthma was classified in well-controlled (60%), partially controlled (31.1%), and uncontrolled asthma (8.9%). A glucose metric was used for the type 1 diabetes group, i.e., the percentage of time in the target range (70–180 mg/dL) (%TIR); it showed that, for almost 60% of the time, on average, the glycemic levels were in the target range. The mean percentage of glycated hemoglobin (%Hba1c) was 7.41%. In the present study, the majority of the diabetic participants showed a percentage of time where glucose was in the optimal range of 59.08%. As to the cancer group, the diagnoses were mainly hematologic tumors (45.5%), but solid tumors (39.4%) and hematologic pathologies (15.2%) were also diagnosed. None of the children was terminally ill and none of them was hospitalized due to cancer at the time of the research. Each child’s mother was recruited. There was a significant difference in their age, assessed using ANOVA; the control sample’s mothers were the oldest. As for the asthma sample, their professions were mainly executive (26.7%); in the cancer sample, most of the mothers were involved in commercial activities and services (21.1%) and in unskilled professions (21.1%); the majority of the type 1 diabetes sample’s mothers were housewives (37.5%); most of the control mothers’ professions was intellectual. A range between 91% and 95% of those respecting the inclusion and exclusion criteria (see Section 2.3) agreed to participate in the study for each subsample. Reasons for refusal were time limits and lack of interest in the research. The sample size needed for a margin error of 8%, with a confidence level of 95%, was 151 participants. The estimated maximum sampling error with a sample size of 171 was 7.49%.

### 2.2. Measures

Ad hoc surveys were created to describe various aspects of the psychological wellbeing of mothers and their respective children. The child version principally included questions about their adaptation during COVID-19 (e.g., their concerns about contagion). The mothers’ version concentrated on sociodemographic information (age and working condition), the worries about the consequences of their child’s contagion due to the chronic illness, and the time (in hours) spent with them on average on a weekday (“time with the child on a weekday (in hours)”). Regarding this last variable, mothers had to make a daily estimation considering the time of compilation, in a contextual situation of gradual reopening and progressively softened movement restrictions, i.e., Phase 4 [3]. The Strength and Difficulties Questionnaire (SDQ) [32,33], used to assess the psychological adjustment of children, is a self-report tool with 25 items, rated on a three-point Likert scale, and organized in five scales: emotional symptoms (EMO), conduct problem (COND), hyperactivity/inattention (HYPER), peer problems (PEER), and prosocial behavior (PROS). The sum of the first four subscales gives a total difficulties score (TDS). It is possible to identify two more factors referred to as internalization (INT) and externalization (EXT) symptoms [33]. The questionnaire has good psychometric properties and has been validated in Italian. Cronbach’s alpha values for the total score (TDS), the internalizing symptoms scale (INT), and the externalizing symptoms scale (EXT) were α(TDS) = 0.71, α(INT) = 0.62, and α(EXT) = 0.63.

The General Health Questionnaire (GHQ-12) [34,35] was used to assess the wellbeing of the mothers in the present study. This psychological screening tool is organized into 12 items (rated on a four-point Likert scale) and identifies minor psychological disorders. Higher scores indicate worse psychological well-being. The questionnaire showed good psychometric properties. Cronbach’s alpha was α(GHQ-TOT) = 0.68.

### 2.3. Procedure

Children with asthma, type 1 diabetes, and cancer were recruited at the Unit of Pediatric Allergy and Respiratory Medicine of the Women and Children’s Health Department (University of Padova), Pediatric Department of Verona Hospital, and Pediatric Oncology of Taranto and Treviso, respectively. For children with asthma, inclusion criteria were age 7 to 15 and a diagnosis of asthma except for severe cases treated with biological drugs; for children with diabetes, inclusion criteria were age 7 to 15 and a diagnosis of type 1 diabetes; for children with cancer, inclusion criteria were age 7 to 15 and a diagnosis of cancer received at least 2 months before, given the severe shock phase that follows the discovery of the disease [36]. For all the clinical samples, exclusion criteria were comorbidity with psychiatric or other chronic conditions and poor knowledge of Italian. The control sample was recruited through snowball sampling with the help of trainees, who were properly instructed. Mothers were phone-called and introduced to the project. For those who agreed, an in-presence compilation session was organized; informed consent for mothers and children was collected, and questionnaires were completed under the supervision of the trainees. All the COVID-19 safety norms were followed. Inclusion criteria were age between 7 and 15, the absence of a diagnosis of any chronic or psychiatric disorder, and poor knowledge of Italian.

For all three clinical samples, a paper-and-pencil survey was administered during the routine visits from May 2020 to September 2020 (Phase 4). Due to the medical condition of the children, they were allowed to have in-presence scheduled control visits at the hospitals. The psychotherapist and the medical staff of the involved wards introduced the research, its aims, and the modalities to all children in line with the inclusion and exclusion criteria and their mothers, who came to the medical visits during the data collection period. Mothers who were willing to join the study had to sign an informed consent for participation. They gave consent for themselves and their children. A specific informed consent was reserved for children older than 12, who had also to sign for themselves if interested in participating. All children were verbally instructed about the study and their consent to join the research was asked. The administration took place in a quiet and separate room in the ward, without interfering with medical procedures. Mothers and children took about 20 min each to fill out the questionnaires. Two ad hoc surveys were administered, one for children and one for mothers, assessing sociodemographic information, questions related to fears about the COVID-19 pandemic, and time spent on average with the child, for mothers, and worries about contagion, for children. Standardized self-report questionnaires were also used to assess children’s psychological adjustment (Strength and Difficulties Questionnaire, SDQ) and mothers’ general wellbeing (General Health Questionnaire, GHQ-12). Moreover, the medical team provided information about the diseases (e.g., time since diagnosis and type of cancer). The project was approved by the Institutional Ethics Committee of Padua (Prot. n. 3671), the Institutional Ethics Committee of Verona (Prot. n. 29097), and the Ethics Committee for Clinical Trials (CESC) (Observational study n. 977/CE). It was performed following the Ethical and Deontological codes of Italian Psychologists.

### 2.4. Statistical Analysis

The normality of the sociodemographic and psychological variables was tested through the Shapiro–Wilk test; most of them were non-normally distributed. The Kruskal–Wallis test (ANOVA) was used for comparisons of the four groups. A multiple linear regression model was computed using children’s internalizing symptoms as the dependent variable. Three dummy variables were created, one for each clinical group, in order to transform the categorical four-level variable “group” into three numeric binary variables; each one took the value of 0 or 1 for the groups with asthma, type 1 diabetes, and cancer. The presence of asthma, type 1 diabetes, and cancer (asthma: yes/no, diabetes: yes/no, and cancer: yes/no), children’s gender, children’s fear of contagion (concerns about contagion), amount of time with mothers in hours, and mothers’ general wellbeing (GHQ-tot) were included as predictors. The assumptions of linearity, no multicollinearity, and homoscedasticity were met and checked through a visual inspection of the correlational matrix for the dependent and independent variables and a residual vs. fitted values plot, respectively. Durbin–Watson’s test indicated no autocorrelation between the model’s residuals (*d* = 1.89). Models’ standard residuals could be considered to approximate a normal distribution. For all the analyses, a significance threshold *p*-value < 0.05 was considered. The SPSS v22.0 software package (SPSS Inc., Chicago, IL, USA) was used for the statistical analysis.

## 3. Results

### 3.1. Differences among Children with Asthma, Type 1 Diabetes, Cancer, and Controls in Sociodemographic and Psychological Variables

As reported in Table 2, significant differences were shown in the item “concerns about contagion”. Higher scores were highlighted in the asthma group, followed by the cancer, type 1 diabetes, and control groups.

As to the SDQ total score, 2.2%, 10.4%, 6.7%, and 0% scored in the clinical range for children with asthma, type 1 diabetes, and cancer, and controls, respectively. No significant differences were found in the total score of the SDQ. Statistically relevant differences emerged in the prosocial behavior subscale with higher scores found in the type 1 diabetes sample, and in the internalizing symptoms scale, in which the highest ratings were recorded in the cancer group.

### 3.2. Differences among Mothers of Children with Asthma, Type 1 Diabetes, Cancer, and Controls in Sociodemographic and Psychological Variables

Regarding the ad hoc survey, mothers’ scores differed significantly in two items (shown in Table 3): “worry about the consequences of the child’s contagion related to the chronic illness” and “time with the child on a weekday”. Higher scores in the first item were reported by the mothers of children with asthma, followed by the mothers of cancer and type 1 diabetes groups. Lastly, mothers of clinically ill children were found to spend more time with their children on weekdays, especially those of the cancer group, followed by those of the type 1 diabetes and asthma groups, compared to mothers of the control group. In the GHQ, the percentage of participants who reported having psychological suffering was 51.1%, 42.3%, 51.5%, and 70.7% for asthma, type 1 diabetes, cancer, and control groups, respectively. No significant differences were found in the total score of the GHQ.

### 3.3. Predictors of Chronically Ill Children’s Internalizing Symptoms

Considering the clinical samples, a linear regression was applied, including as the dependent variable the internalizing symptoms scale of the SDQ. This model showed the diagnosis of cancer or type 1 diabetes as significant positive predictors and underlined the variable of the survey “time with the child on a weekday” as a significant negative predictor (model fit measures: *F* = 2.799; *p* < 0.050, Adj. R^2^ = 0.066). Therefore, being affected by cancer, suffering from type 1 diabetes, and spending less time with mothers seemed to be associated with an increase in children’s internalizing problems (see Table 4).

## 4. Discussion

The present research aimed to explore the role of time spent with mothers on children with chronic diseases’ psychological wellbeing, during Phase 4, comparing three clinical samples of children with asthma, type 1 diabetes, cancer, and their mothers with a control group of healthy children and mothers. Firstly, the three clinical samples were compared to the control group in their psychological wellbeing. Moreover, an explorative comparison of children and mothers of the three chronic conditions was made. The psychological impact of the pandemic has mainly been studied in healthy populations, while less is known about chronically ill children and their caregivers [3,22,23].

As for children of all groups, they showed similar psychological adjustment, consistent with studies claiming that no differences in psychological wellbeing were evidenced during the pandemic [3,24]. The present sample children were not psychopathological. However, with respect to children’s concerns of the contagion, significant differences were found. Both children with asthma and cancer showed higher rates in this variable compared to control and type 1 diabetes participants, consistent with the hypothesis [3,24]. The asthma group’s major concerns about contagion may be due to the specific pathology’s characteristics. Given that the COVID-19 syndrome and asthma have similar symptoms, children of this group may have been more worried that an eventual contagion could be more dangerous and life-threatening for them [37]. At that time, the COVID-19 medical effects on children with asthma were still unknown, while it has more recently been reported that people with asthma are not at greater risk for COVID-19 contagion, nor for more severe outcomes in case of infection [38]. The same explanation may be given for the greater concerns for contagion reported by children with cancer: the more physical vulnerability, the more dangerous the exposure to the contagion risk; thus, major values of concern may be registered [39,40]. Moreover, the oncological patients received the diagnosis more recently, giving more uncertainty regarding the disease course. Lastly, the asthmatic group’s mothers reported spending less time with their children compared to the other clinical groups; this may also have had an impact on children’s worries.

Differences were found in the SDQ subscale “prosocial behavior”, with higher rates obtained by the type 1 diabetes group compared to the cancer one. The SDQ’s prosocial scale assesses the resources of children connected to their kindness and helpfulness, their willingness to share, and the interpersonal abilities, which allow them to take actions that benefit life together [41,42]. The lower scores recorded in the cancer group compared to the type 1 diabetes one may reflect particular aspects of being affected by cancer; these patients are usually isolated from others because of immunosuppression, contributing to the lack of social interactions, especially in pandemic times [22]. As to the internalizing domain (SDQ), children with cancer highlighted higher scores with respect to those with asthma and their healthy peers. These greater values may have emerged in association with the specific experiences linked to cancer; invasive medical procedures, stressful separations for possible hospitalizations [43], and specialized medications jointly with the distressful COVID-19 emergency may contribute to a more intense experience of the pandemic [44,45]. Moreover, children with severe asthma and type 1 diabetes were not included in the study.

When it comes to the mothers, the present results are partially coherent with the hypothesis. It was found that asthma and cancer groups’ mothers scored higher than the type 1 diabetes group’s mothers in relation to the worry about the consequences of their children’s contagion. The literature has shown that, during the pandemic, the trend for chronically ill children’s parents was a worsening of their psychosocial health [26,27]. Mothers of this group may have been more worried as an eventual contagion could be more dangerous and life-threatening for their children, given the similarities to the COVID-19 syndrome, as discussed earlier [37]. As to the cancer group, mothers may have been more worried considering the physical vulnerability due to immunosuppression [22]. Actually, patients with tumors are more susceptible to severe complications and mortality in the case of COVID-19 contagion [46]. Consistent with our results, Hullman et al. (2010) found that children with asthma are perceived as more vulnerable by their parents, if compared with those with type 1 diabetes or cancer, due to the unpredictability of pulmonary symptoms, less frequent medical monitoring, and the greater risk for emergency department visits [28]. It seems that this pattern occurred also during Phase 4. These results suggest the importance of assessing illness-related worries in order to give appropriate information and help mothers and children of these populations to cope with these fears, especially during pandemic times.

Consistent with the hypothesis, mothers of children with chronic illnesses reported spending more time with them if compared to the control group, especially mothers of the type 1 diabetes and cancer groups [5]. As for type 1 diabetes, this may have been due to the need for glycemic level monitoring and insulin administration, which require close supervision [27]. As for cancer, this may have been due to children’s vulnerability due to a severe diagnosis, which was pronounced more recently and to which they needed to adapt [36]. It is curious that the asthma group’s mothers were not those who spent more time with children, given that they reported being more worried about the consequences of the child’s contagion. This aspect needs to be further studied, as asthma onset and outcomes can be related to psychological features such as the mother–child relationship [47]. It would be interesting to know how mothers and children spent time together; they may have been involved in activities, sharing affective moments [7], or they may have been more controlling and anxious for monitoring and treatment administration, given the changing in health services availability [8]. Moreover, the family size or the presence of other important people may have influenced the quality of this time together; for example, in some families, grandparents may have moved into the house to help with the parenthood duties [48]. Furthermore, mothers of all the groups did not show significant differences in their psychological general wellbeing, not consistent with the hypothesis, as it was expected for mothers of chronically ill children to be more compromised [26,27]. The majority of mothers in all groups reported having psychological suffering. It may be thought that they were more impaired by the harshness of caring for a child during the COVID-19 pandemic, regardless of whether their child was healthy or chronically ill, and regardless of a difference among the three pathologies.

When it comes to the third hypothesis, the linear regression model showed that being affected by cancer, suffering from type 1 diabetes, and spending less time with mothers seemed to be associated with an increase in children’s internalizing problems. As hypothesized, time with mothers seems to have protected children’s psychological wellbeing during Phase 4 [6,7]. No studies, to our knowledge, have explored the role of time with parents for chronically ill children during the pandemic. The literature claims that, for healthy children and adolescents, time with parents was considered a benefit in COVID-19 home quarantine [6]. As said before, it has not been explored how children and mothers spent time together. Mothers are usually the main caretakers when it comes to their children’s chronic illness [49]; it may be thought that the time spent together was also for controlling and administering medications. However, it seems that the time with mothers itself is a resource for healthy and chronically ill children’s psychological wellbeing. These children had the opportunity to be closer to their mothers and may have been reassured by their presence, not feeling alone with their disease. Mothers may have been a source of information and emotional containment to help them give meaning to the specific situation they were living in and better manage their illnesses. Lastly, it is curious that having asthma was not a significant predictor; this result may also be associated with the fact that mothers in the asthmatic group reported spending less time with their children if compared with other groups’ mothers. As gender was not a significant predictor in the model, the bias evidenced by the literature regarding greater time spent by mothers with their daughters seemed not to be present in the study’s samples [18,19].

### 4.1. Limitations

The research presented some limitations linked to the characteristics of the samples, particularly the small number of participants and the nonhomogeneous number of people in each yield, which made it difficult to generalize the results. Regarding the clinical participants, those with more severe diseases were excluded from the clinical samples. Moreover, medical data accounting for the control of each disease were not assessed as possible predictors of a worsening of internalizing symptoms. Furthermore, no data regarding time since diagnosis were collected for the asthma group. Although it would have been interesting to compare data collected in COVID-19 and pre-COVID-19 periods, we did not have a baseline psychological pre-COVID evaluation for the present sample. Thus, the present study concentrated on Phase 4. Moreover, it was not possible to consider the vaccine status of the participants as vaccines were still not available at the time of the data collection [50]. The research was geared only toward the time mothers spent with children and did not evaluate the involvement of fathers or other important family figures. Furthermore, how time was spent with the mothers was not explored. Lastly, most of the variables were assessed through self-report questionnaires; although the SDQ and GHQ are considered valid standardized psychological tools, they can be influenced by social biases, such as social desirability.

### 4.2. Future Research and Clinical Implications

Further research may focus on deepening knowledge about the role of time spent with mothers for chronically ill children. Firstly, as the perception of time passing is a subjective variable, it would be interesting to compare mothers’ and children’s estimation of time spent together, including an item about the time spent with mothers, as well as for the children’s groups. Further studies may more deeply comprehend the specific vision of time in children, according to its particular characteristics (such as being brought up by both parents, the number of siblings, with whom the child lives, the age of the mother at the moment of birth, the type of chronic disease, and its genetic background) [51,52]. Future perspectives may investigate which kinds of activities seem to be shielding factors. Moreover, it would be interesting to distinguish the time spent in daily activities from disease management and understand the type of parental medical attention given to the child’s chronic disease (hyper-control, monitoring, or mere support). With a view to prevention, it could be essential to identify the children’s social resources and promote spending time with significant others (i.e., mothers, referring to the present study) in fragile situations.

Phase 4 was studied in the present research to assess if time with mothers was a shielding factor in shifting to normality contexts (not properly in emergencies). The topic of time spent with mothers in chronically ill children during the pandemic was not previously studied at our knowledge. Moreover, the present work may give useful information for prevention and health promotion interventions, to be implemented in times such as those we are living in, of gradual shifting to normality. The present study actually has some clinical implications. Preventative, clinical programs or parent training may be established to support the proper management of time with chronically ill children; parents could be helped to balance the time spent in medical assistance and in pleasant activities. Therefore, not only may interventions based on time spent together be important in terms of variability, but this optic may also lead to a generalization of the importance of time spent with significant persons in the more general clinical practice.

## 5. Conclusions

The present study emphasized the importance of time during the pandemic; the presence and, thus, the time taken to stay in presence with other significant people was seemingly identified as an important resource for psychological wellbeing during Phase 4. The long time spent in the pandemic’s strict lockdown showed the fundamental importance of time management. The awareness of the essentiality of the administration of time may be employed in constructive and meaningful activities, in order to provide and improve the quality of life. Therefore, both in great situations of stress caused by emergency situations and in times of gradual shifting to normality, a reflection can be made on how to spend time. Benefit finding can be an involved method in demonstrating the advantages of the pandemic experience and of being together [25,26,27,28,30]. Most of the studies regarding the COVID-19 pandemic frequently focused on the causes pf increased difficulties during the pandemic and the discomfort of the emergency. The optics of the benefit finding set the cruciality of the research in a positive light; this construct may also drive research in this field by promoting the resources, instead of just underlying the problems.

## Figures and Tables

**Table 1:** Demographics and descriptive variables for the four samples. ANOVA was used for assessing statistically significant differences between groups with respect to age and mothers’ working situation, and Student’s *t*-test was used for assessing statistically significant differences between groups with respect to the time since the diagnosis.

Children		Asthma (*n* = 45)		Type 1 Diabetes (*n* = 52)		Cancer (*n* = 33)		Control (*n* = 41)		*F*/*t*-Test	*p*-Value
		Mean and Percentages	SD	Mean and Percentages	SD	Mean and Percentages	SD	Mean and Percentages	SD		
Age		10.67	2.28	10.84	2.17	11.12	3.15	11.02	2.25	2.275	>0.050
Gender (M/F)	Males	77.8%		53.8%		48.5%		68.3%		3.275	<0.050
	Females	22.2%		46.2%		51.5%		31.7%			
Time since the diagnosis (in years)				5.34	3.05	1.86	1.03			7.544	0.001
**Mothers**		**Asthma** **(*n* = 45)**		**Type 1 Diabetes** **(*n* = 52)**		**Cancer** **(*n* = 33)**		**Control** **(*n* = 41)**		***F*/*t*-Test**	***p*-Value**
Age		43.93	5.30	43.48	5.69	41.76	6.21	45.61	5.42	2.918	<0.050
Working situation	Intellectual professions	22.2%		5.3%		19.5%		39.0%		2.341	>0.050
	Technical professions	8.9%		10.5%		12.5%		14.6%			
	Executive professions	26.7%		10.5%		15.7%		9.8%			
	Commercial activities and services	13.3%		21.1%		14.1%		7.3%			
	Artisans, laborers	2.2%		18.4%		3.2%		0.0%			
	Unskilled laborers	2.2%		13.2%		15.6%		12.2%			
	Unskilled professions	24.4%		21.1%		19.4%		17.1%			

**Table 2:** Kruskal–Wallis test for the clinical and control samples. Cohen’s f values: small effect size (0.10), medium effect size (0.25), and large effect size (0.40). Post hoc tests using Bonferroni correction are reported. A = asthma group, D = type 1 diabetes group, O = cancer group sample; C = control group; n, the sample size for each group; SD, the standard deviation; *p*, the *p*-value; Cohen’s f, the appropriate measure of effect size.

Variable		Group	*n*	Mean	SD	Mean Rank	Test Statistic	*p*	Cohen’s f	Post Hoc
Child	Concerns about contagion	Asthma	45	1.91	0.596	102.01	18.921	<0.001	0.28	A > C A > D O > C O > D
		Type 1 diabetes	52	1.51	0.543	74.70				
		Cancer	33	1.90	0.790	97.92				
		Control	41	1.41	0.547	67.33				
	SDQ—prosocial behavior	Asthma	45	8.98	1.971	89.70	11.954	<0.010	0.35	D > O
		Type 1 diabetes	52	9.12	1.875	96.91				
		Cancer	33	7.87	1.821	63.27				
		Control	41	8.41	1.549	74.28				
	SDQ—internalizing symptoms	Asthma	45	2.80	2.242	73.62	9.543	<0.050	0.42	O > A O > C
		Type 1 diabetes	52	3.63	2.811	87.97				
		Cancer	33	4.43	2.979	102.92				
		Control	41	2.66	1.797	72.78				

**Table 3:** Kruskal–Wallis test for the clinical and control samples’ mothers. Cohen’s f values: small effect size (0.10), medium effect size (0.25), and large effect size (0.40). Post hoc tests using Bonferroni correction are reported. A = asthma group, D = type 1 diabetes group, O = cancer group sample; C = control group; n, the sample size for each group; SD, the standard deviation; *p*, the *p*-value; Cohen’s f, the appropriate measure of effect size.

Variable		Group	*n*	Mean	SD	Mean Rank	Test Statistic	*p*	Cohen’s f	Post Hoc
Mother	Worry about the consequences of the child’s contagion related to the chronic illness	Asthma	45	2.38	0.576	72.18	10.087	<0.010	0.24	A > D O > D
		Type 1 diabetes	52	2.00	0.663	53.13				
		Cancer	33	2.38	0.554	71.83				
		Control	–	–	–	–				
	Time with the child on a weekday (in hours)	Asthma	45	7.24	4.973	68.42	19.483	<0.001	0.75	O > C D > C
		Type 1 diabetes	52	9.88	5.329	96.78				
		Cancer	33	10.38	6.087	96.60				
		Control	41	6.34	3.732	61.43				
	GHQ—total	Asthma	45	18.00	4.592	88.68	3.577	>0.050	0.31	
		Type 1 diabetes	52	16.73	4.239	76.76				
		Cancer	33	18.36	4.885	96.91				
		Control	41	17.76	2.764	86.00				

**Table 4:** Linear regression model of children’s internalizing symptoms. Bold for *p*-values < 0.050. *B*, unstandardized beta; SE, standard error; Beta, standardized beta; t, the *t*-value (the coefficient divided by its standard error); 95% CI, confidence intervals; Adj. R^2^, adjusted R-squared.

Predictor	*B* (95% CI)	SE	Beta	*t*	*p*
Intercept	1.651 (−0.494; 3.796)	1.085		1.521	>0.050
Asthma: yes/no	0.134 (−913; 1.181)	0.530	0.025	0.253	>0.050
Diabetes: yes/no	1.236 (0.182; 2.290)	0.533	0.230	2.317	**<0.050**
Cancer: yes/no	1.770 (0.496; 3.043)	0.644	0.267	2.747	**<0.050**
Child’s gender	0.630 (−0.170; 1.429)	0.405	0.126	1.556	>0.050
Child’s concerns about contagion	0.266 (−0.370; 0.901)	0.322	0.070	0.826	>0.050
Time with the child on a weekday	−0.076 (−0.152; 0.000)	0.038	−0.163	−1.968	**<0.050**
GHQ—total	0.016 (−0.074; 0.106)	0.045	0.028	0.351	>0.050

## Data Availability

Data are available from the corresponding author (S.S.) on reasonable request.
